# Patient-specific signaling signatures predict optimal therapeutic combinations for triple negative breast cancer

**DOI:** 10.1186/s12943-023-01921-9

**Published:** 2024-01-16

**Authors:** Heba Alkhatib, Jason Conage-Pough, Sangita Roy Chowdhury, Denen Shian, Deema Zaid, Ariel M. Rubinstein, Amir Sonnenblick, Tamar Peretz-Yablonsky, Avital Granit, Einat Carmon, Ishwar N. Kohale, Judy C. Boughey, Matthew P. Goetz, Liewei Wang, Forest M. White, Nataly Kravchenko-Balasha

**Affiliations:** 1 The Institute of Biomedical and Oral Research, The Hebrew University of Jerusalem, 9103401 Jerusalem, Israel; 2 Department of Biological Engineering, Massachusetts Institute of Technology, Cambridge, MA 02139 USA; 3 Koch Institute for Integrative Cancer Research, Massachusetts Institute of Technology, Cambridge, MA 02139 USA; 4 Tel Aviv Sourasky Medical Center, Tel Aviv, Israel; 5 Sackler Faculty of Medicine, Tel Aviv University, Tel Aviv, Israel; 6 Sharett Institute of Oncology, Hebrew University-Hadassah Medical Center, 9103401 Jerusalem, Israel; 7 Department of Surgery, Samson Assuta Ashdod University Hospital, Ashdod, Israel; 8 Department of Surgery, Mayo Clinic, Rochester, MN 55905 USA; 9 Department of Oncology, Mayo Clinic, Rochester, MN 55905 USA; 10 Department of Molecular Pharmacology and Experimental Therapeutics, Mayo Clinic, Rochester, MN 55905 USA

**Keywords:** Personalized combined treatment, EGFR, Targeted therapy, Triple-negative breast cancer, Phosphotyrosine proteomics, Information theory, Patient-specific signaling signatures

## Abstract

Triple negative breast cancer (TNBC) is a heterogeneous group of tumors which lack estrogen receptor, progesterone receptor, and HER2 expression. Targeted therapies have limited success in treating TNBC, thus a strategy enabling effective targeted combinations is an unmet need. To tackle these challenges and discover individualized targeted combination therapies for TNBC, we integrated phosphoproteomic analysis of altered signaling networks with patient-specific signaling signature (PaSSS) analysis using an information-theoretic, thermodynamic-based approach. Using this method on a large number of TNBC patient-derived tumors (PDX), we were able to thoroughly characterize each PDX by computing a patient-specific set of unbalanced signaling processes and assigning a personalized therapy based on them. We discovered that each tumor has an average of two separate processes, and that, consistent with prior research, EGFR is a major core target in at least one of them in half of the tumors analyzed. However, anti-EGFR monotherapies were predicted to be ineffective, thus we developed personalized combination treatments based on PaSSS. These were predicted to induce anti-EGFR responses or to be used to develop an alternative therapy if EGFR was not present.

In-vivo experimental validation of the predicted therapy showed that PaSSS predictions were more accurate than other therapies. Thus, we suggest that a detailed identification of molecular imbalances is necessary to tailor therapy for each TNBC. In summary, we propose a new strategy to design personalized therapy for TNBC using pY proteomics and PaSSS analysis. This method can be applied to different cancer types to improve response to the biomarker-based treatment.

**Supplementary Information:**

The online version contains supplementary material available at 10.1186/s12943-023-01921-9.

## Background

Targeted therapies have revolutionized breast cancer treatment. However, triple-negative breast cancer (TNBC), lacking estrogen receptor, progesterone receptor, and HER2 expression, still relies on chemotherapy.

Recent clinical trials have shown promise with targeted therapies, such as anti-PARP inhibitors or immunotherapies, increasing median progression-free survival of TNBC patients by several months [1]. Additional efforts to implement anti-TNBC targeted therapies have included kinase inhibitors, such as EGFRi [2]. Although EGFR is highly expressed in many TNBC patients [3], clinical trials have not demonstrated significant beneficial effects [4]. Lack of response or resistance to treatment can be caused by, for example, inherent resistance or adaptive mechanisms [4–6]. Due to TNBC heterogeneity, patient stratification based on activated signaling networks should be considered to improve sensitivity to targeted therapies.

We addressed this substantial problem by designing personalized treatments for patient-derived TNBC tumors. Using information theory-based computational analysis [7] of tyrosine phosphorylation signaling networks [8, 9], we computed a set of distinct ongoing processes, named patient-specific signaling signatures (PaSSS), in 28 TNBC patient-derived tissues to predict individualized treatments. This combined experimental and computational approach enabled prediction of patient-specific monotherapy or combination therapy for each PDX tumors. While EGFR was found as a central target in over 50% of the cases, treatment with EGFR *monotherapies* was predicted to be ineffective in most cases. In vivo validation of predicted mono- and combination therapies highlights the predictive power of this approach, which can be used for various cancer types.

## Results

### Phosphotyrosine signaling networks of TNBC tissues are highly heterogeneous

We choose to quantify phosphotyrosines (~ 200–400 pY in each sample), since pY are primary targets for approved or in-development targeted drugs.

Our observation revealed significant patient-to-patient heterogeneity, consistent with prior research (Fig. S1A, B).

The heterogeneity was not only reflected by dissimilar signaling pathways characterizing different TNBC patients, but also by heterogeneity within the pathways. BR98 and PDX11 had high pEGFR but low pMAPK1/3, while BR45, PDX6 and PDX16 had high pMAPK3 but low pEGFR (Fig. S1B, S2). Intra-pathway heterogeneity was not restricted to the EGFR/MAPK pathway alone. Upstream RTKs did not necessarily correlate with downstream targets, like low pKIT in BR98 or pMUC1 in PDX2, and high levels of their downstream pSTAT3 (Table S1, S2), or high pMUC1/pKIT and low pSTAT3 in PDX6 (Table S3). Unique evolutionary pathways can be caused by genomic changes in TNBC tumors, which split/alter signaling pathways (Fig. S1C).

### PaSSS procedure to discover changes in signaling flux

**Fig. 1:**
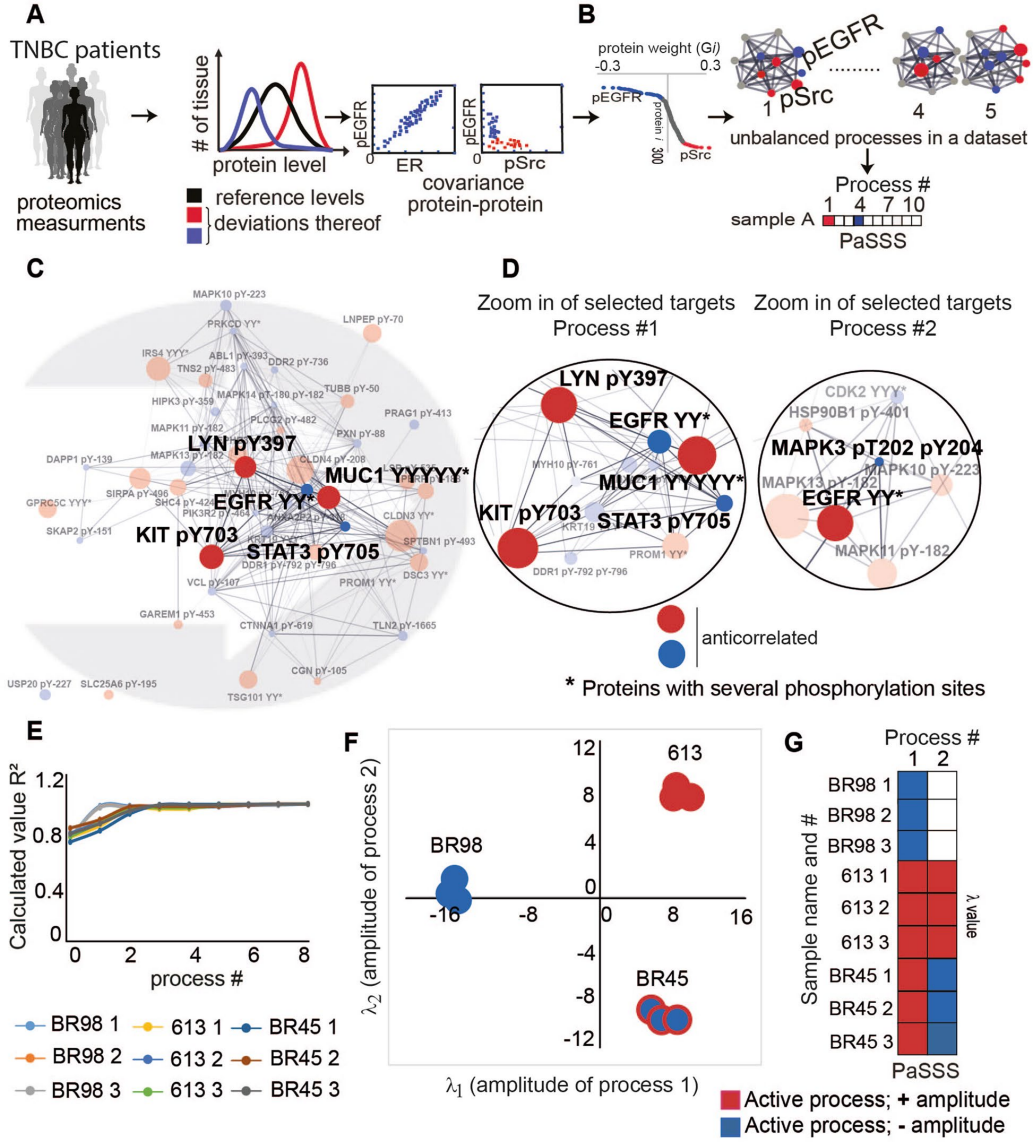
PaSSS analysis of TNBC patient-derived samples. **A** Steps of the analysis: Proteins whose phosphorylation levels deviate from the reference (steady) state in the same or opposite directions (left panel) are identified via co-variance matrix and surprisal analysis (exemplified by pEGFR, ER and pSrc, right panel). Here pEGFR and ER deviate in the same direction from the steady state, whereas pEGFR and pSrc are orthogonal. **B** Protein weights (G*i*) are quantified (Supplementary Methods). Proteins located on the tails of G*i* plots are co-expressed and grouped into unbalanced processes using STRING protein–protein connections and G*i.* For each sample, PaSSS is assigned to represent only sample-specific processes, e.g., with significant amplitude ${\lambda }_{\alpha }\left(k\right)$ (all steps of the analysis are detailed in Supplementary Methods). **C**-**D** Processes found in BR45, BR98 and 613 TNBC subgroup: see process 1, full in (**C**) and zoomed *in* in (**D**). Zoomed *in* images of processes 1 and 2 representing this subgroup are shown in (**D**). Proteins with multiple phosphosites include MUC1 (YYYYY: pS1207-pY1212, pY1212, pY1203, pY1209 and pY1209-pY1212), EGFR (YY: pY1172 and pY1197) in process 1, and EGFR (YY: pY1172 and pY1197) in process 2. **E** *2 processes* were sufficient to reproduce this subset and thus provide the full characterization of the data **(**Supplementary Methods). **F** Mapping of BR45, BR98 and 613 tissues in 2D space using ${\lambda }_{\alpha }\left(k\right)$ values. The sign of ${\lambda }_{\alpha }\left(k\right)$ determines the location on the map in terms of sample separation in nD space (Supplementary Methods). Sample location in (**F**) is schematically converted into a “barcode” (**G**). Red-labeled proteins in (**D**) are upregulated in the red-labeled processes in “PaSSS” barcodes (**G**), whereas blue-labeled proteins are upregulated in blue-labeled processes (Supplementary Methods)

Environmental and genomic constraints can prevent a biological system from achieving steady state. Tumors with unique functional properties will be affected by different constraints. PaSSS analysis uses a thermodynamic-based surprisal analysis [10] to identify individualized constraints causing changes in protein/phosphorylation levels from the steady state. Experimental levels of each protein are decomposed into steady state values (values unaffected by constraints) and deviations due to constraints (Fig. 1A). Proteins that deviate from steady-state in a coordinated manner are classified as unbalanced processes. Multiple (n) unbalanced processes can be found in each dataset. Not all processes are active in every tumor; patients may have one or more unbalanced processes, which reflect *the patient-specific signaling signature* (PaSSS [7], Fig. 1B). Thus, each tumor is represented by a PaSSS *combination of processes* in nD space. For therapeutic efficacy, the tumor's signaling flow must be decreased by inhibiting central targets from each active process in the tumor.

The PaSSS analysis approach is illustrated using the BR45, BR98, and 613 PDX subset (Fig. 1 and Supplementary Methods). PaSSS analysis revealed two ongoing processes in this subset; highlighted subsections of these processes are presented in Fig. 1D and show selected positively and negatively correlated proteins. Process 1 suggests that if pEGFR is induced, pKIT is decreased and vice versa.

PaSSS analysis confirms the strong decoupling between pEGFR and pMAPK3 and reveals that process 1 affects pEGFR but not MAPK3. In process 2, the proteins are inversely related, suggesting that inhibiting one protein will not affect the EGFR/MAPK pathway.

Processes 1 and 2 were enough to represent the variability between the samples in the BR45/BR98/613 subset (Fig. 1E). The samples' variability can be visualized using process amplitudes in 2D space: BR98 has one non-zero coordinate—process 1. BR45 and 613 have two unbalanced processes (Fig. 1F). The positive sign of process 1 in BR45 and 613 is opposite to that of BR98: meaning that proteins upregulated in BR98 are downregulated in BR45/613 tumors and vice versa. Additional process 2 separates BR45 and 613 tumors in 2D. Orthogonal deviation from steady state in BR45 and 613 results in mapping along different Y-axis directions.

The 2D map is transformed into a barcode, displaying PaSSSs as square groups indicating coordinates in 2D (Fig. 1G). Red or blue colors indicate important processes, while inactive processes are shown in white. Visualizing more than 3 dimensions is difficult, so this representation is useful when mapping samples in higher-dimensional space. The barcodes' colors aid in understanding protein regulation in each sample. For instance, pEGFR increases in BR98 due to process 1, while pKIT decreases in this tissue (Fig. 1D,G).

### TNBC tumors are characterized by a patient-specific combination of ~ 2 unbalanced processes

The PaSSS analysis revealed that TNBC tumors displayed 1–4 unbalanced processes each, averaging 2 per tumor, with central proteins including pKIT, pEGFR, pSrc family proteins, and PI3K (Fig. 1G, S3, S4; Tables S1-S6). Despite a clear heterogeneity of TNBC tumors, namely each tumor harbored a different PaSSS, the suggested targeted therapy for each patient is simple and should include ~ 2 drugs capturing the entire PaSSS imbalance (Tables S1-S6).

Only 3 out of 28 tumors may benefit from anti-EGFR monotherapy. 12 tumors may benefit from a combination therapy including EGFR, such as EGFRi and KIT/MUC1i. The other tumors are expected to benefit from non-EGFRi combined therapies, such as MAPK1/3, Src family kinases, IGFR1, or MUC1 inhibitors.

### PaSSS provides efficient targeted therapy for TNBC

**Fig. 2:**
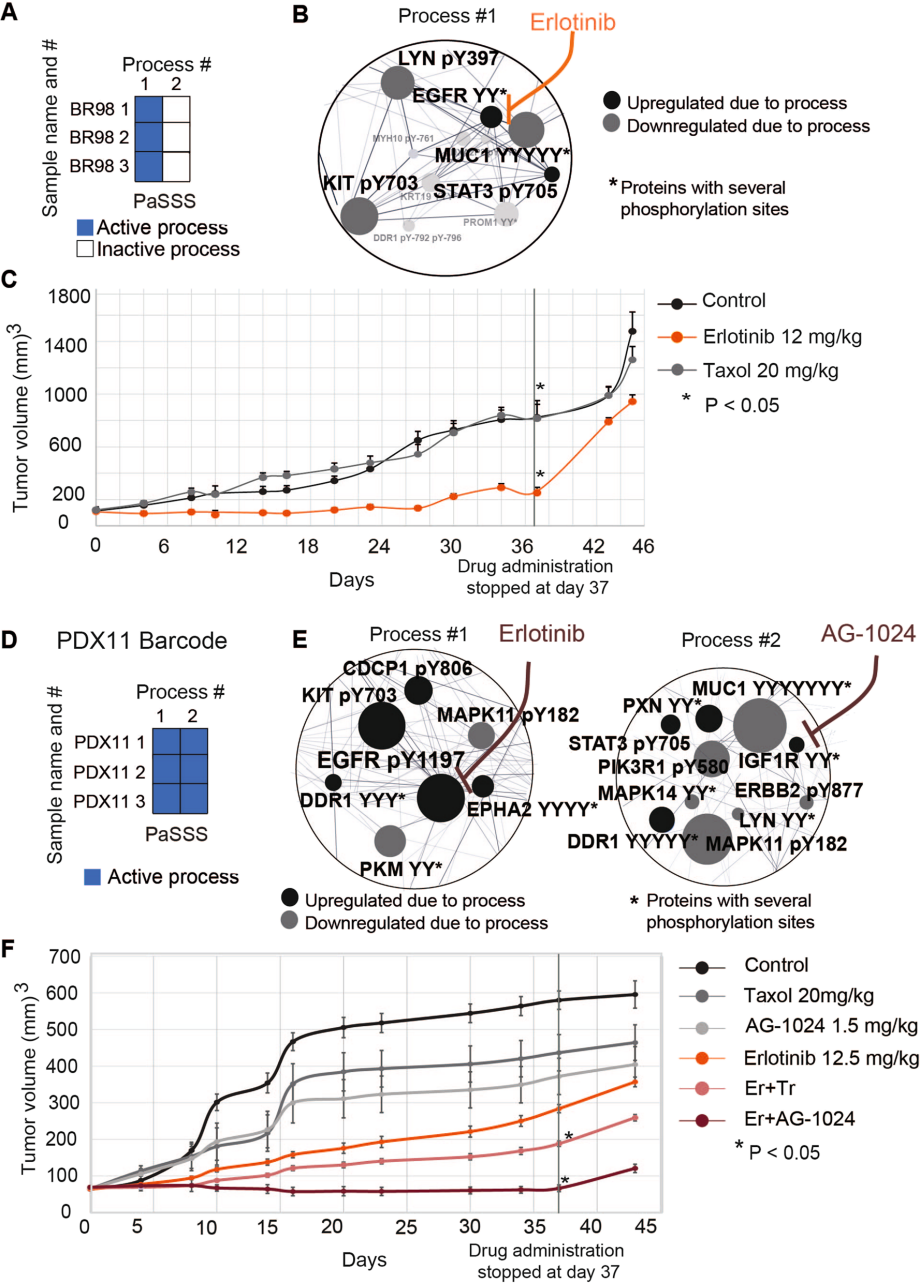
PaSSS-based therapy surpasses clinically prescribed Taxol and is patient-specific. **A** Barcode representing basal PaSSS of BR98 tumor, namely the set of active unbalanced processes. BR98 harbors one process, process 1. **B** Zoom-in image of the unbalanced process 1 active in BR98. The complete list of proteins that participate in the process 1 can be found in Table S1. Black circles denote upregulated proteins due to the process (as calculated using a product ${G}_{i\alpha }{\lambda }_{\alpha }\left(k\right)$, Supplementary Methods), gray circles represent downregulated proteins in the same process. Functional connections are according to STRING database. **C** BR98 tissues were orthotopically transplanted into NSG mice at the age of 6–7 weeks. Once tumors reach 60–80 mm^3^ volume, PaSSS predicted therapy (Erlotinib) and paclitaxel were used to assess the response of the tumors to these therapeutic strategies. At day 37 the treatment was stopped and tumor growth continued to be monitored. **D** Barcode representing basal PaSSS of PDX11. **E** Zoom-in image of the unbalanced processes active in PDX11 as well as the predicted drugs targeting the central proteins in each process. The complete list of proteins that participate in each of the processes can be found in Table S3. Black circles denote upregulated proteins due to the process (as calculated using a product ${G}_{i\alpha }{\lambda }_{\alpha }\left(k\right)$), gray circles represent downregulated proteins in the same process. Functional connections are according to STRING database. **F** PDX11 tissues were orthotopically transplanted as described in **C**. The experimental groups included control (vehicle) group, erlotinib monotherapy group, AG-1024 monotherapy group (anti-IGFR), Taxol chemotherapy, Erlotinib (Er) + AG-1024 (PaSSS therapy) and Er + Trametinib (Tr) (combination predicted to be less effective). Treatments were carried out for 36 days. On day 37 the treatments were stopped and tumors were grown untreated

To validate therapeutic predictions by PaSSS, we selected TNBC tumors BR98 and PDX11. PaSSS analysis suggests that EGFR inhibitor monotherapy is effective for BR98 (Fig. 2A, B) but not for PDX11. Instead, therapy for PDX11 should target both pEGFR and pIGFR-centered processes (Fig. 2D, E).

In-vivo experiments confirm that PaSSS predicted treatment decreased tumor growth relative to Paclitaxel (used in clinics) or other treatments (Fig. 2C, F, S5, S6).

PaSSS analysis of *treated* tissues (Fig. S7, Table S7) showed that the EGFR-centered process 1 was reduced in erlotinib-treated BR98 samples. Process 2 differentiated between control and paclitaxel-treated samples. Stress-related proteins were induced due to process 2 in paclitaxel-treated tumors, however it was insignificant in erlotinib-treated samples.

Induced pEGFR and pIGFR subnetworks were found in PDX 11 in two distinct processes, processes 1 and 2, confirming previous analysis of basal levels. Their decrease was observed with PaSSS, Erlotinib + AG-1024 treatment (Fig. S8), indicating a stronger effect of combined therapy. No change in process 2 was observed with monotherapies. Additionally, a new process 3 emerged in samples that received monotherapies. Certain proteins like pKIT, pMAPK3, and pEGFR showed increased levels in response to AG-1024. Similarly, pPI3K, pKIT, and pABL were increased in response to erlotinib. These processes may contribute to treatment resistance. When the samples were given the double therapy of erlotinib and trametinib (*not predicted* by PaSSS), another developing process, process 5, was detected. This process included proteins like pPI3K, pMAPK3, and pMAPK1 (Table S8).

IGF1R and EGFR-centered processes responded as expected to PaSSS treatment, but cell movement/cell adhesion proteins were activated, potentially as an adaptive response or due to small subpopulations being selected. However, these proteins did not cause drug resistance during the study period as the PaSSS treatment effectively stopped PDX11 growth.

## Discussion

Recent advances in subgrouping TNBC [11] indicate potential for alternative therapies beyond cytotoxic chemotherapy [12]. However, besides some limited efficacy, targeted monotherapies failed in the clinic. Computational approaches such as machine/deep learning methodologies [13] for classifying TNBC do not yet allow for prescribing patient-specific targeted drug combinations. These approaches may not accurately detect rare features in TNBC patients [14]. Failure to address all *patient-specific* features may result in treatment resistance.

To overcome this limitation, we integrated quantitative phosphotyrosine proteomics with information-theoretic PaSSS analysis to provide a tailored treatment strategy. PaSSS analysis identified a patient-specific *set of ongoing processes* in each tissue, which were examined to find targetable proteins. PaSSS treatment has the important benefit of being tailored against processes rather than specific oncomarkers. As long as it captures the entire PaSSS a physician can design several optional drug combinations (Supplementary Methods). Future preclinical platforms [15] validating PaSSS drug synergy can replace in-vivo experiments.

PaSSS drug combinations outperform standard care and non-predicted combined therapies. Tumor development was suppressed, although a potential adaptive response or subpopulation selection was seen in PDX11. Examining biopsy specimens before and during therapy in clinical setting can evaluate drug effectiveness and identify adaptive response pathways or new aggressive subpopulations.

EGFR remains a critical target in many TNBC patients [4]. However, drugs that target the EGFR pathway, alone or with chemotherapy, had limited benefit [4]. Our study confirmed the clinical trial findings, indicating that EGFR monotherapies will have a low response rate (< 10%) and suggested tailored combined therapies to induce anti-EGFR response. PDX11 represents a subset of these patients. Other PaSSS treatments may not include EGFRi.

In summary, we propose a novel approach combining pY proteomics and PaSSS analysis to design personalized treatments for TNBC. This strategy can be applied to various cancer types.

### Supplementary Information

**Additional file 1:** Supplementary Figures (Figures S1-S8).

**Additional file 2.** Supplementary materials and methods.

**Additional file 3. Tables S1-S8**.

## Data Availability

All data associated with this study are present in the paper or the Supplementary Materials.

## Abbreviations

EGFR: Epidermal Growth Factor Receptor
IGF1R: Insuline-like Growth Factor Receptor
MAPK: Mitogen-Activated Protein Kinase
KIT: KIT Protooncogene, receptor tyrosine kinase
STAT3: Signal transducer and activator of transcription 3
MUC1: Mucin 1, cell surface associated
PI3K: Phosphatidylinositol 3-kinase
EGFRi: EGFR inhibitor
SA: Surprisal Analysis
Her2: Human epidermal growth factor receptor 2
cMet: Cell surface mesenchymal-epithelial transition factor
TNBC: Triple Negative Breast Cancer
PaSSS: Patient specific signaling signature
PDX: Patient-derived xenograft
