# Supplementary material for: Patient-specific signaling signatures predict optimal therapeutic combinations for triple negative breast cancer
Source: Mol Cancer. 2024 Jan 16;23:17. doi: 10.1186/s12943-023-01921-9 (PMC10790458; doi:10.1186/s12943-023-01921-9)
Supplement: Supplementary file 1 — Additional file 1: Supplementary Figures (Figures S1-S8). [file 12943_2023_1921_MOESM1_ESM.pdf]

Supplementary Figures for

Patient-specific signaling signatures predict optimal therapeutic combinations for triple negative breast cancer

Heba Alkhatib †, Jason Conage-Pough *et al.* †
† Contributed equally to this work

*Corresponding authors: Nataly Kravchenko-Balasha, natalyk@ekmd.huji.ac.il, Forest White, fwhite@mit.edu

The file includes:

Figs. S1 to S8

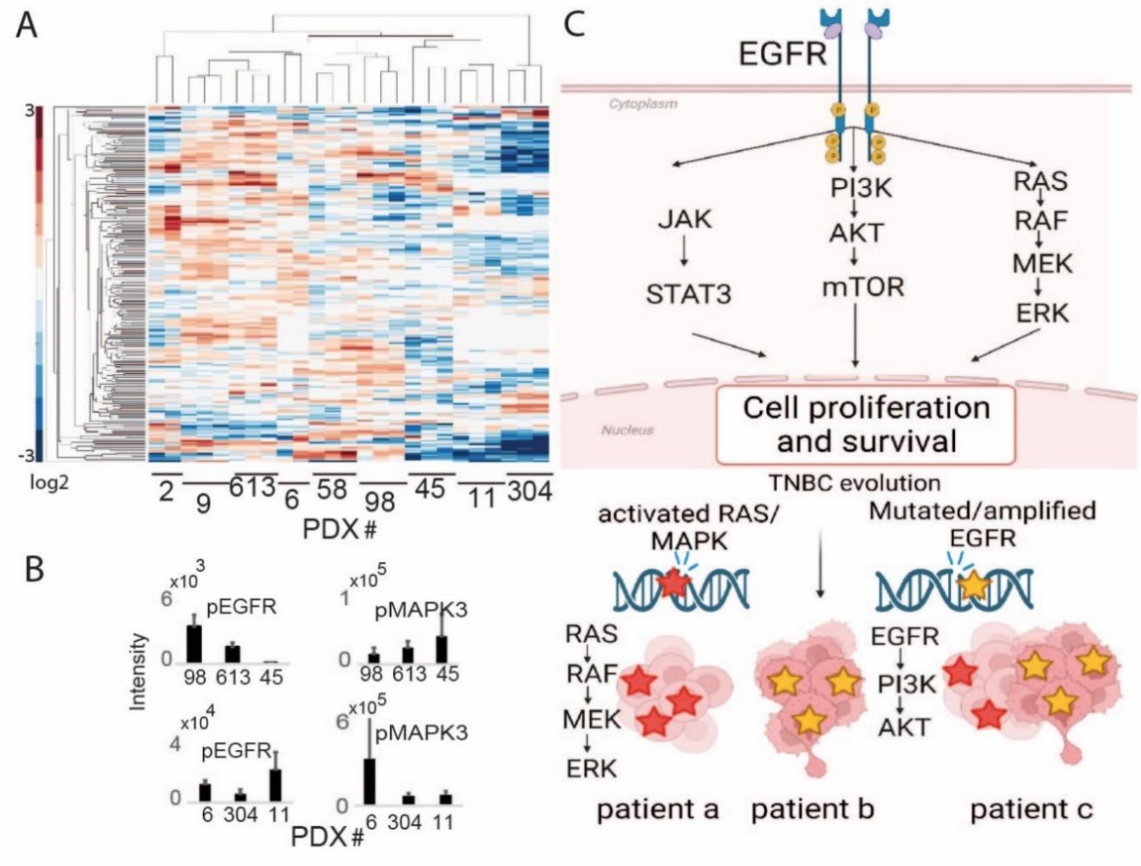

Fig. S1 pTyr proteomics reveals significant inter-tumor heterogeneity (**A**) 28 tumors (47 samples) were collected from either Hadassah or Mayo clinic (Patient cohort, Supplementary Materials and Methods). Heatmap representation of experimental phosphotyrosine (pY) proteomics of 9 (in triplicates except for PDX2 and PDX6 which were duplicates) patient-derived tumors collected in Hadassah hospital. Tables S1 through S3 provide raw data. Tables S4-6 provide data from tumors collected at the Mayo Clinic. (**B**) Examples for decoupling of EGFR/MAPK signaling (EGFR-pY1172 and MAPK3-pT202-pY204 in PDXs # 98, 613 and 45 (upper panel) and in PDXs #6,304 and 11 (lower panel). The experimental levels of pEGFR and pMAPK3 are shown. (**C**) Schematic representation of examples of EGFR decoupling at the patient population or cellular (intra-tumor) levels.

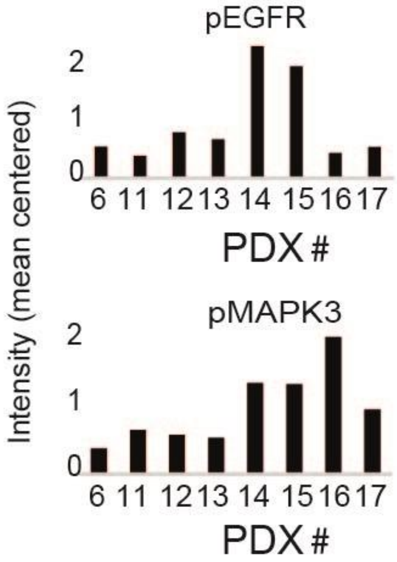

Fig. S2. Examples for decoupling of EGFR/MAPK signaling (EGFR pY1172 and MAPK3(pY204) in PDXs from Mayo clinic's dataset. Tables S4 through S6 provide raw data. pMAPK3(pY204) and pEGFR (pY1172) are co-induced in PDX14, in contrast to uncorrelated behavior of pMAPK3 (pY204) and pEGFR (pY1172) in PDX16 (Table S5).

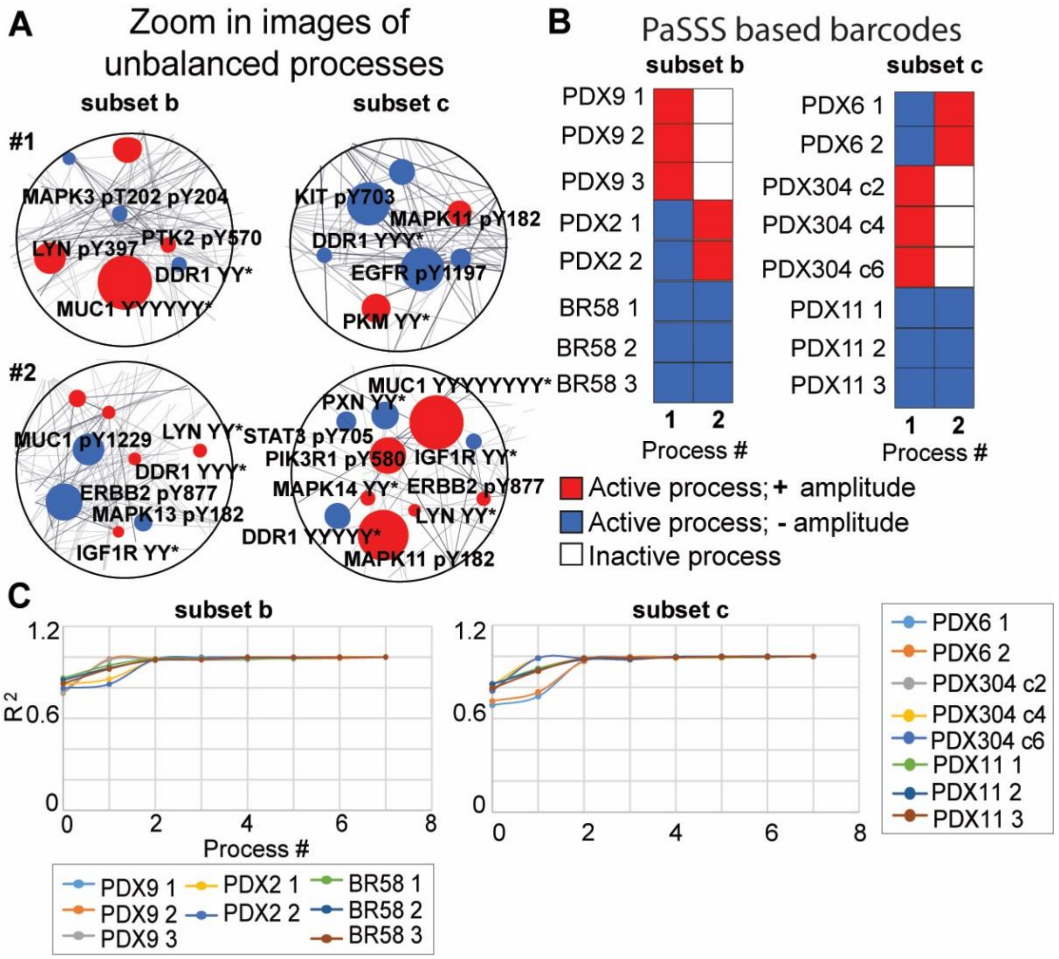

Fig. S3. PaSSS analysis of subset b and c of PDXs (PDX9, PDX2 and BR58) and (PDX6, PDX304, PDX11) datasets with the predicted therapeutic targets (Tables S2, S3). (**A**) Example of zoomed in images of unbalanced processes obtained using PaSSS analysis and suggesting the protein targets for each PDX tissue. Proteins with multiple phospho-sites include Muc1 is phosphorylated on 6 sites (YYYYYY: pS1207-pY1212, pY1212, pY1203-pY1212, pY1209-pT1211, pY1191 and pY1229), DDR1 is phosphorylated on 2 sites (YY: pY792 and pY796) in process 1 subset b, LYN is phosphorylated on 2 sites (YY: pY508 in two genes), DDR1 is phosphorylated on 3 sites (YYY: pY792, pY792-pY797 and pY796) and IGF1R is phosphorylated on 2 sites (YY: pY1161 and pY1165) in process 2 of subset b, DDR1 is phosphorylated on 3 sites (YYY: pY520, pY792 and pY796-pY797) and PKM is phosphorylated on 2 sites (YY: pY105 and pY370) in process 1 subset c, MUC1 is phosphorylated on 8 sites (YYYYYYYY: pS1207-pY1212, pY1212, pY1203, pY1203-pY1212, pY1209, pY1209-pY1212, pY1229 and pY1191), PXN is phosphorylated on 2 sites (YY: pY88 and pY118), IGF1R is phosphorylated on 2 sites (YY: pY1161 and pY1165), MAPK14 is phosphorylated on 2 sites (YY: pT180-pY182 and pY182), LYN is phosphorylated on 2 sites (YY: pY397 and pY508) and DDR1 is phosphorylated on 5 sites (YYYYY: pY513, pY520, pY792, pY792-pY796 and pY796-pY797) in process 2 of subset c. The red proteins are upregulated due to positive amplitude of the process and the blue proteins are upregulated due to negative amplitude of the process (shown in **B**). For example; a suggested target for PDX 9 is MUC1 (representing process 1, subset b); for PDX2 (subset b) are MAPK3 and IGF1R; for PDX 11 (subset c) are EGFR and IGF1R and so on (Tables S2 and S3). Central proteins that have a strong connection with other proteins within the network and FDA approved inhibitors are considered potent candidates targets. The connection between proteins is based on STRING

database (**B**) The identified PaSSS barcodes are presented showing the active processes for each tumor replicate with their respective amplitude. (**C**) R^2 values suggesting the number of significant unbalanced processes important in a given subset. When the values converge or reach a plateau, the number of the significant unbalanced processes is determined.

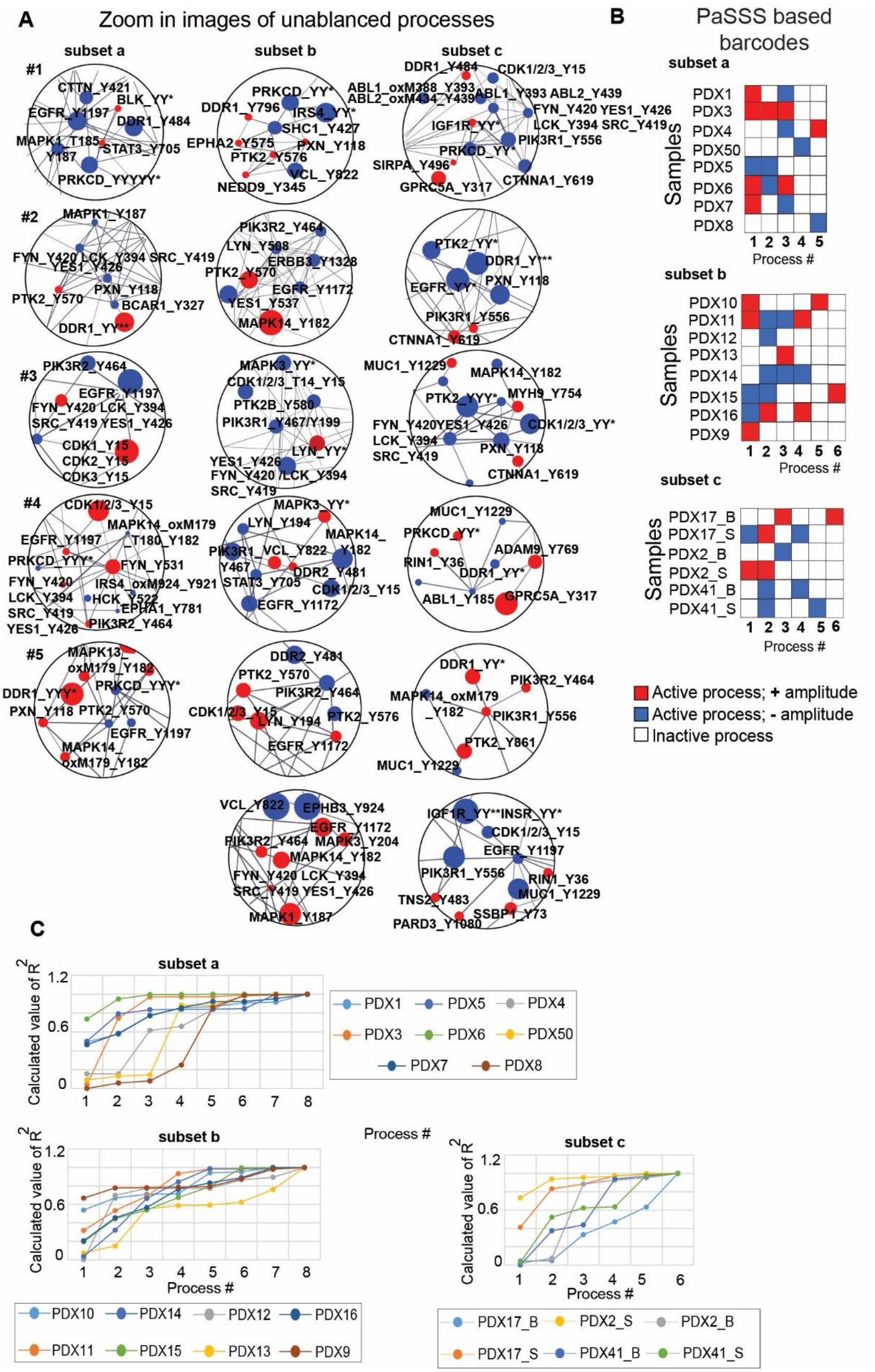
A
Zoom in images of unablanced processes
subset a
subset b
subset c
#1
#2
#3
#4
#5
B
PaSSS based barcodes
subset a
PDX1
PDX3
PDX4
PDX50
PDX5
PDX6
PDX7
PDX8
Samples
Process #
subset b
PDX10
PDX11
PDX12
PDX13
PDX14
PDX15
PDX16
PDX9
subset c
PDX17_B
PDX17_S
PDX2_B
PDX2_S
PDX41_B
PDX41_S
Active process; + amplitude
Active process; - amplitude
Inactive process
C
Calculated value of R2
Process #
PDX1
PDX5
PDX4
PDX3
PDX6
PDX50
PDX7
PDX8
PDX10
PDX14
PDX12
PDX16
PDX11
PDX15
PDX13
PDX9
PDX17_B
PDX2_S
PDX2_B
PDX17_S
PDX41_B
PDX41_S

Fig. S4. PaSSS analysis of three Mayo clinic's subsets of PDX with the predicted therapeutic targets (Tables S4-S6)**. (A**) Example of zoomed in images of unbalanced processes obtained using PaSSS analysis and suggesting the protein targets for each PDX tissue. The red proteins are upregulated due to positive amplitude of the process and the blue proteins are upregulated due to negative amplitude of the process (as shown in **B**). Central proteins that have a strong connection with other proteins and FDA approved inhibitors are considered potent candidates for targeting. The connections shown between proteins are STRING based. (**B**) The identified PaSSS barcodes are presented showing the active processes for each tumor sample with their respective amplitude. (**C**) R^2 values suggesting the number of significant unbalanced processes important in a given subset. When the values converge or reach a plateau, the number of the significant unbalanced processes is determined.

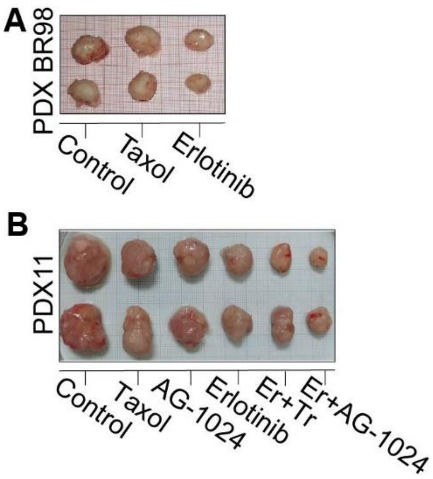

Fig. S5. Representative, treated and untreated BR98 and PDX11 tumors, harvested after 36 days respectively, are shown.

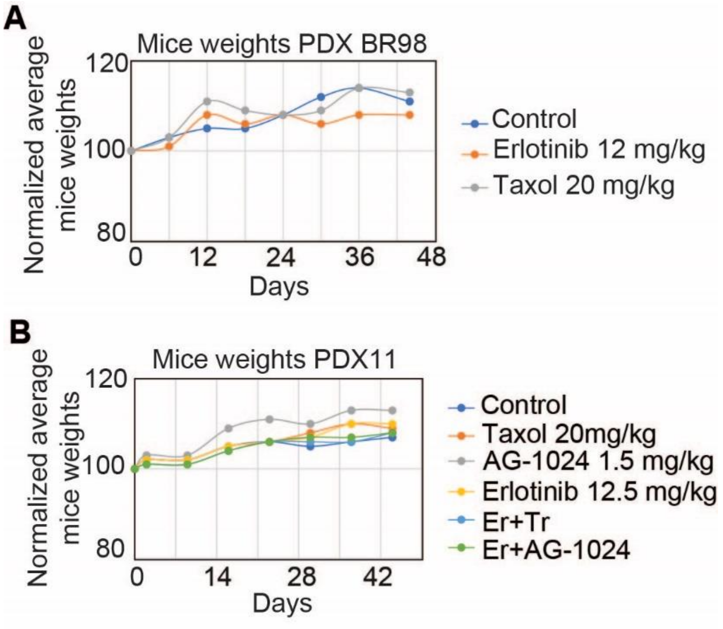

Fig. S6. Normalized average mice weights (values are divided by the first weight of each mouse at the beginning of the experiment) for BR98 and PDX11 (Hadassah subset). Experiments for each group per given day are shown.

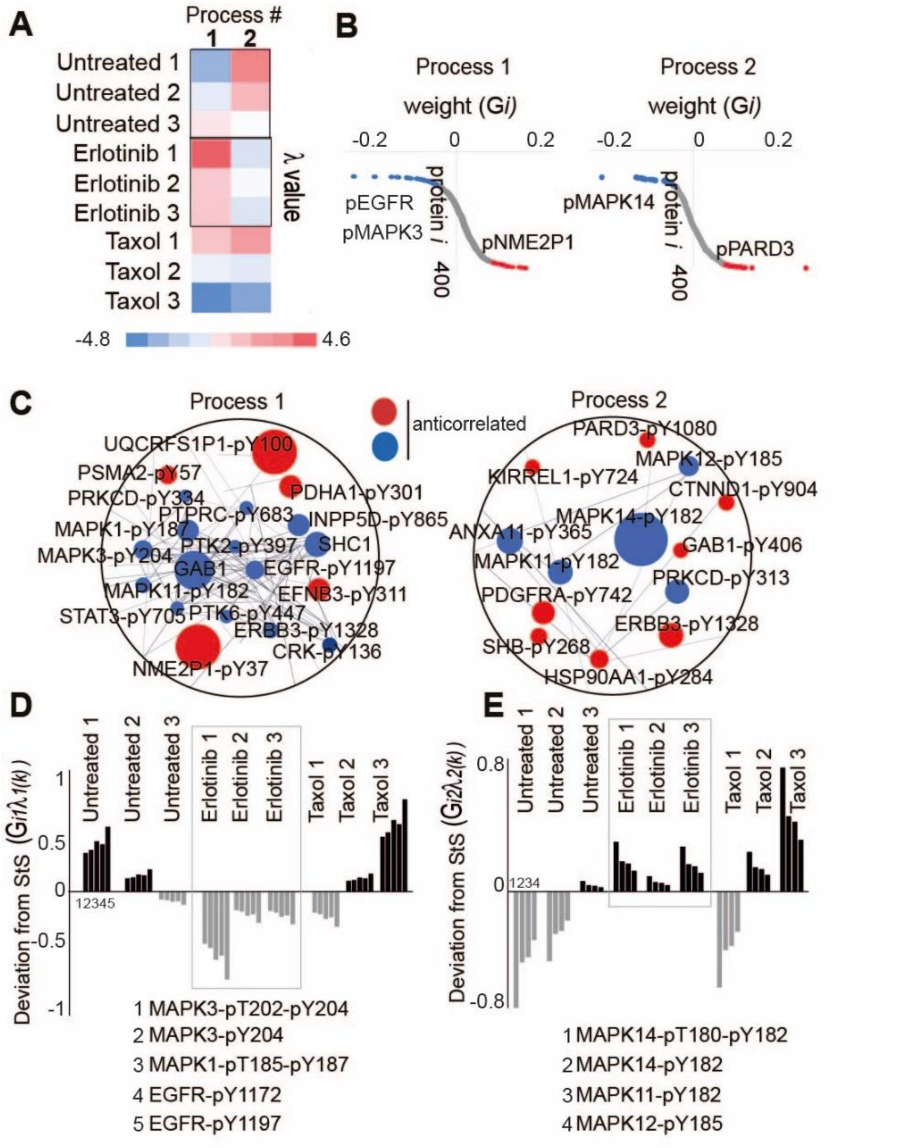

Fig. S7 PaSSS analysis of BR98 samples *treated* with PaSSS therapy or paclitaxel. (A) BR98 tissues were orthotopically transplanted into NSG mice at the age of 6-7 weeks. Once tumors reach 60-80 mm^3 volume, predicted therapy and paclitaxel were used to assess the early response. At day 5 tumors were resected and subjected to pTyr phosphoproteomics. (**A**) A heatmap showing the amplitudes $\lambda_\alpha(k)$ of the two most dominant processes, 1 and 2, in the dataset **(Table S7).** $\lambda_\alpha(k)$ sign determines the correlation or anti-correlation between *the same* processes in different samples **(B)** Sorted protein weights (G*i*) are quantified and presented for the processes 1 and 2. G*i* values located on the tails in (B) along with a probability for functional connections (STRING) are used to illustrate the unbalanced processes in (**C**). (**C**) Zoomed in images of processes 1 and 2 are shown. Blue proteins are upregulated in the blue-labeled processes in **(A)** (**e**.g. pEGFR is upregulated in untreated samples, however downregulated in erlotinib treated samples, Methods) however downregulated in red-colored processes in **(A).** Panels **(D-E)** demonstrate this point. **(D-E)** The product of the protein weight and the process amplitude, $G_{i\alpha}\lambda_\alpha(k)$ is shown for selected proteins from process 1 (**D**) or process 2 (**E**). The product indicates the extent of deviation in expression level of a protein *i* from its reference state (StS) due to process α in a sample *k* (e.g pEGFR in untreated samples in process 1). Positive values of $G_{i\alpha}\lambda_\alpha(k)$ indicate an increase relative to the steady state in a sample *k*, and negative values indicate reduction.

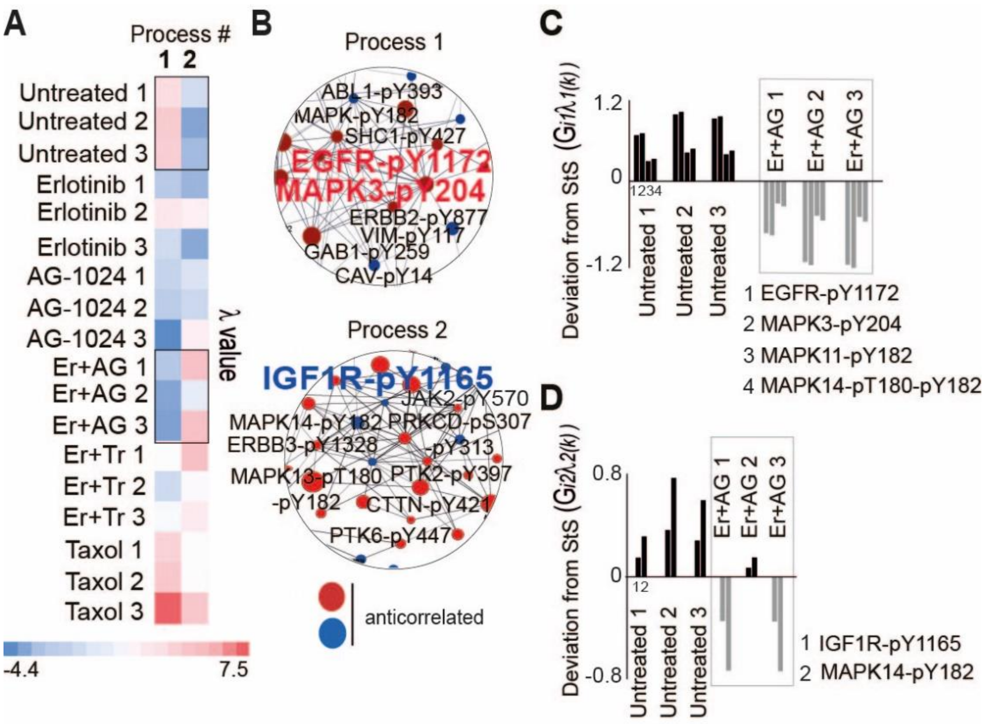

Fig. S8 PaSSS analysis of PDX11 samples *treated* with PaSSS therapy, monotherapies or paclitaxel. (A) PDX11 tissues were orthotopically transplanted into NSG mice at the age of 6-7 weeks. Once tumors reach 60-80 mm^3 volume, Erlotinib (Er), AG-1024 (anti-IGFR, AG), PaSSS Combination of Erlotinib and AG-1024 (Er+AG), combination predicted for another tumor (Er+Tr (trametinib)) or Paclitaxel (Taxol) were used to assess the early response. At day 5 tumors were resected and subjected to pTyr phosphoproteomics. (**A**) A heatmap showing the amplitudes $\lambda_\alpha(k)$ of the two most dominant processes, processes 1 and 2. Additional emerging processes, characterizing samples treated with monotherapies or with Er+Tr, are shown in Table S8. (**B**) Zoomed in images of processes 1 and 2 are shown. Red-colored proteins are upregulated in the processes labelled in red in (**A**) (e.g pEGFR is upregulated in process 1 in untreated samples however downregulated in Er+AG samples) and blue proteins are upregulated in the blue-labeled processes in (**A**) (e.g., pIGF1R in process 2 in untreated samples, but downregulated in Er+AG, Methods). Panels (**C-D)** exemplify this point. (**C-D**) The product of the protein weight and the process amplitude, $G_{i\alpha}\lambda_\alpha(k)$ are shown for selected proteins from process 1 (**C**) or process 2 (**D**). The product indicates the extent of deviation in expression level of a protein i from its reference state (StS) due to process α in a sample k (e.g pEGFR in untreated samples in process 1). Positive values of $G_{i\alpha}\lambda_\alpha(k)$ indicate an increase relative to the steady state in a sample k, and negative values indicate reduction. Although IGF1R and EGFR signaling responded as predicted in response to PaSSS treatment with AG-1024 and erlotinib, proteins associated with cell movement/cell adhesion were induced - see for example Cav, VIM in *process 1*, and PTK2, CTNND1 in *process 2*.
