# Supplementary material for: Patient-specific signaling signatures predict optimal therapeutic combinations for triple negative breast cancer
Source: Mol Cancer. 2024 Jan 16;23:17. doi: 10.1186/s12943-023-01921-9 (PMC10790458; doi:10.1186/s12943-023-01921-9)
Supplement: Supplementary file 2 — Additional file 2. Supplementary materials and methods. [file 12943_2023_1921_MOESM2_ESM.pdf]

Supplementary Materials and Methods for

Patient-specific signaling signatures predict optimal therapeutic combinations for triple negative breast cancer

Heba Alkhatib †, Jason Conage-Pough *et al.* †
† Contributed equally to this work

*Corresponding authors: Nataly Kravchenko-Balasha, natalyk@ekmd.huji.ac.il, Forest White, fwhite@mit.edu

Materials and Methods

Computational methods

Surprisal analysis

The computational method is founded on the idea that biological systems—including tumors—follow quantitative rules similar to those of nonequilibrium, open chemical systems [1]. We postulate that, subject to environmental and genetic constraints, the biological system approaches a state of lowest free energy (maximum entropy) at the usual conditions of a given temperature and pressure [2]. These constraints prevent the tissue from reaching a steady state. A steady state in biology is one in which all state variables have constant values or where inputs and outputs are balanced [3]. As a result, we should be able to characterize it and deviations thereof by experimental measures (when many patients/conditions or time points are compared). To identify the most stable states in the high throughput datasets (such as proteomics obtained from multiple patients) we apply a maximum entropy-based surprisal analysis (SA)[1, 4]. SA's derived equation (for details see [1]) allows us to compute the steady-state expression levels (when constraints are 0) and then deviations from these values for each protein: $\underbrace{X_i(k) =}_{\text{exp}\, levels\, of\, protein\, i} \underbrace{X_i^0(k)}_{steady\, state\, levels} \exp(-\underbrace{\Sigma_{\alpha=1} G_{i\alpha}\lambda_\alpha(k)}_{deviation\, due\, to\, constraint/s}$ **[eq 1]**. Proteins deviating from the steady state in a similar manner (co-expressed) are grouped into **unbalanced processes** **(Fig. 1A-B** of the main text and[5–7]). $G_{i\alpha}$ **is a weight (importance) of a protein *i*** in each constraint (= unbalanced process) α and $\lambda_\alpha(k)$ is an amplitude of the process α in each sample/cell k.

In practice, we calculate the covariance matrix of natural logarithms of protein expression levels as dictated by the theory [1] and then fit it into eq. 1 to calculate the expected protein levels at steady-state and deviations from that level in all investigated samples. Matrix containing the natural logarithm of proteins is used as an intermediate step, which calls for the construction of two square, symmetric, co-variance matrices. One is smaller with a maximal rank equivalent to the number of samples (used to compute $\lambda_\alpha(k)$ values) and the second is larger, equivalent to the number of proteins (used to compute $G_{i\alpha}$ values). Consequently, the maximum number of processes we may get cannot be greater than the rank of the smallest matrix and thus cannot be greater than the number of samples [1].

Using Singular Value Decomposition (SVD) these matrices are diagonalized to calculate eigenvectors and eigenvalues. Eigenvectors and eigenvalues are used to calculate the

amplitudes of the processes: $\lambda_\alpha(k)$ for each sample and $G_{i\alpha}$ values (a very detailed procedure can be found in reference [1]).
Importantly, we fit the experimental data accurately by incorporating as few processes as possible until they adequately reproduce the experimental data (**Fig. 1E of the main text**). Thus, if we discover that we have less unbalanced processes than the rank of smallest matrix - this indicates that the dataset can be reliably reproduced and characterized and that its size is sufficient (e.g. there are enough samples to find all possible constraints in the examined dataset). If this is not the case, the dataset should be increased in size.
Only proteins with significant $G_{i\alpha}$ values (located on the tails of the $G_{i\alpha}$ distribution plots) are grouped into unbalanced processes (**Fig. 1B).** We present the processes as networks where circle radiuses represent protein weights ($G_{i\alpha}$) and edges the probabilities for protein-protein interactions based on literature [8]. $\lambda_\alpha(k)$ is **an amplitude of the process α in each tissue/sample *k*** [1]. Only processes that exceed the error limit, computed as explained in [5] are included in a patient-specific set of unbalanced processes named patient-specific signaling signatures (**PaSSS**, see the sections "**Determination of the number of significant unbalanced processes"** and **"Patient-specific signaling signatures (PaSSS) calculation"** for more details.
Simultaneously, each patient's tumor may have several unbalanced processes - on average two in this study (computed as detailed in the section below **"Patient-specific signaling signatures (PaSSS) calculation"**.
All calculated $\lambda_\alpha(k)$ and $G_{i\alpha}$ values are provided in Supplementary **Tables S1-S8 (**tabs "Lambda", "G" correspondingly) for *all* tumor samples obtained from either Hadassah or Mayo Clinic's Medical Centers.
The samples were divided into several subsets due to a couple of factors. Some of the PDX tumors were obtained from the Mayo Clinic in Rochester, MN and others were obtained from Hadassah Hospital in Jerusalem. PDX tissues were acquired over several years, with some of the Mayo Clinic specimens being acquired and analyzed several years earlier than the samples from Hadassah Hospital. Moreover, even within these subsets, the number of PDX tumors often exceeded the multiplex capability of TMT-multiplexing, and thus samples had to be analyzed in separate analyses.
We investigated each dataset separately for two reasons: 1) not to lose proteomic information (merged datasets resulted in the loss of ~50% of available phospho-sites and thus less accurate proteomic characterization); and 2) to see if the outcomes of independent studies were equivalent.

Signs of $G_{i\alpha}$ and $\lambda_\alpha(k)$

The sign $G_{i\alpha}$ as presented in all figures indicates *the correlation or anti-correlation* between proteins in the same process. For example, in a certain process α , proteins can be assigned the values: $G_{protein1,\alpha}$ = -0.06, $G_{protein2,\alpha}$ = 0.07, and $G_{protein3,\alpha}$ = 0.00, indicating that this process altered proteins 1 and 2 in opposite directions (i.e. protein 1 is upregulated and protein 2 is downregulated, **or vice versa** due to the process α), while not affecting protein 3. Note that each protein can take part in a number of unbalanced processes at once. In the manuscript, proteins with negative $G_{i\alpha}$ values are always labeled in **blue**, whereas proteins with positive $G_{i\alpha}$ values are always labeled in **red**.
Importantly, not all processes are active in all tumors. The term $\lambda_\alpha(k)$ represents the importance of the unbalanced process α in the tumor *k*. Its sign indicates the correlation or anti-correlation between the same processes in different tumors. For example, if the process α=1 is assigned the values: $\lambda_1(BR98)$ = -15.8, and $\lambda_1(613)$ = 8.015 (Table S1), it means that this process influences the tumors of the patients indexed BR98 and 613 in the opposite direction.

Calculation of protein upregulation/downregulation using a product $G_{i\alpha}\lambda_{\alpha}(k)$

To discriminate between correlated and anti-correlated phosphopeptides and to specify the degree to which a protein i is impacted by process α, weights for each phosphopeptide ($G_{i\alpha}$) were utilized. The product of the protein weight and the process amplitude, $G_{i\alpha}\lambda_{\alpha}(k)$ indicates the extent of deviation in expression level of a protein i from its reference state due to process α in a sample k. Positive values of $G_{i\alpha}\lambda_{\alpha}(k)$ indicate an increase relative to the steady state in a sample k, and negative values indicate reduction.
The algorithm for calculating the amplitudes of the unbalanced processes and $G_{i\alpha}$ values is presented in **Table 1**. A detailed step by step mathematical procedure of SA can be found in supplementary file of Vasudevan et al[5]. Phosphoproteins with significant weights $G_{i\alpha}$ (located on the tails of the plots representing sorted $G_{i\alpha}$ values, Supplementary **Tables S1-S8**) are grouped using STRING [9] (see below) to provide a biological interpretation of each process.

Determination of the number of significant unbalanced processes

We analyze the number of processes required to replicate the experimental data as previously described[5, 10] in order to determine the number of significant processes in the dataset. We plotted $\Sigma G_{i\alpha}\lambda_{\alpha}(k)$ for $\alpha = 1, 2, \ldots, n$ against ln $X_i(k)$ for different proteins, i, and for different values of n, and examined the correlation (R^2) between them as n was increased. An unbalanced process, $\alpha = n$, was considered significant if it improved the correlation significantly relative to $\alpha = n - 1$.
The values of 1% of the most stable proteins in (those with lowest standard deviations) were used to establish the threshold limits for $\lambda_{\alpha}(k)$. In order to create a patient-specific barcode and calculate a deviation from steady state, only processes that exceed the threshold limitations were taken into account.

Calculation of patient-specific signaling signatures (PaSSS) and assignment of tailored therapy.

Unbalanced processes with amplitudes which exceeded threshold limits were included in the patient-specific barcodes. Threshold limits for $\lambda_{\alpha}(k)$ values are calculated as previously described [5, 6]. $\lambda_{\alpha}(k)$ ($\alpha = 1, 2, 3\ldots$ n) and then parameterized as follows: for each α, if $\lambda_{\alpha}(k) >$ error limit then it is parameterized to 1; if $\lambda_{\alpha}(k) < -$ error limit then it is parameterized to -1; and if $-$error limit $< \lambda_{\alpha}(k) <$ error limit then it is parameterized to 0 (**Tables S1-S6,** tabs "Barcodes and treatments"). In figures 1 and 2 of the main text PaSSS barcodes are presented as square groups. Squares representing processes with non-zero $\lambda_{\alpha}(k)$ values are labeled by red or blue squares.

Based on available drugs (e.g., FDA approved) and druggable targets in each process, each PaSSS was allocated **a drug combination**. Anti-cancer drugs were sourced from [11]. Importantly, the PaSSS approach suggests which processes should be targeted rather than a particular onco-marker. As long as the entire PaSSS imbalance is captured, a clinician is free to choose a protein target in each process, preferably one with many protein-protein linkages, while taking into account a variety of factors such as cost, low toxicity in the drug therapy combination (which may occur due to drug-drug interaction), or availability of a drug targeting several processes at the same time. Less selective targeted drug may be more effective since it might capture several PaSSS processes at the same time.

Furthermore, building preclinical platforms [12] to assess PaSSS therapies and their predicted synergy may help to speed up the adoption of PaSSS treatments in clinics.

Unbalanced process subnetwork generation

The STRING database[9] was used to define functional connections between phosphoproteins which were found to be influenced by the unbalanced processes (proteins located on the tails of $G_{i\alpha}$ plots as illustrated in Fig. 1B of the main text). Visualizations of subnetworks based on STRING parameters were generated using Cytoscape [13] software. The functional relationships between the phosphoproteins that were discovered to be influenced by the unbalanced processes were defined using the STRING database [9]. Using the Cytoscape [13] software, visualizations of subnetworks depending on STRING parameters were produced.

Table 1 Algorithm (used in MATLAB) for identifying the amplitude of the unbalanced processes ($\lambda_\alpha(k)$) and the weight of the proteins ($G_{i\alpha}$) using SA. A detailed mathematical procedure is available at [1].

**Identification of unbalanced processes amplitudes ($\boldsymbol{\lambda_\alpha(k)}$) and protein weights ($\boldsymbol{G_{i\alpha}}$)**
**Input: phosphoproteome (I = 1, …, n),**<br>[G,W,V] = svd(log(Data));<br>rows = size(Data,1);<br>columns = size(Data,2);<br>if rows>columns<br>L=V*W(1:columns,:);<br>end<br>if rows<columns<br>W0 = zeros(columns-rows,columns);<br>WW=[W; W0];<br>L = V*WW;<br>end<br>**Output: G =** $G_{i\alpha}$**, L =** $\lambda_\alpha(k)$

Experimental methods

Patient cohort

Patient cohorts were obtained from two hospitals: Hadassah hospital (Tables S1-S3) and Mayo clinic (Tables S4-S6) - 28 TNBC tumors (47 different samples as some of them were analyzed in triplicates or duplicates).

Samples from the Mayo Clinic have been described in a previous publication[14]. The samples from the Hadassah hospital are discussed below:

BR45: Patient derived tumors were established from a triple negative invasive lobular breast cancer female patient. The tissue was obtained from the local chest wall recurrence, s\a mastectomy, chemotherapy and radiotherapy. After implantation into the NSG mice, the tissue formed tumors that were used for the *in vivo* experiments.

BR98: Patient derived tumors were established from a TNB metastatic invasive duct carcinoma female patient. Cells were derived from pleural effusion. No treatment was applied at the time of the fluid extraction. The patient was previously treated with chemotherapy and radiotherapy. After cells were injected into mice, the developed tumors were used for the *in vivo* experiments.

BR58: Patient derived tumors were established from an ovary serous papillary carcinoma female patient with breast and lung metastasis. The tissue was obtained by breast biopsy. At this time, patient was treated with chemotherapy and radiotherapy. After cells were injected into mice, the developed tumors were used for the *in vivo* experiments.

PDX11: Patient derived tumors were established from a triple negative invasive ductal carcinoma from a female patient. The tissue was obtained from the mastectomy. At the time the sample was obtained, neoadjuvant chemotherapy treatment was already initiated. After implantation into mice, the tissue formed tumors that were used for the *in vivo* experiments.

PDX9: Patient derived tumors were established from a triple negative invasive ductal carcinoma from a female patient. The tissue was obtained from the mastectomy. After implantation into mice, the tissue formed tumors that were used for the *in vivo* experiments.

PDX6: Patient derived tumors were established from a triple negative metastatic invasive ductal carcinoma from a female patient. The tissue was obtained by breast biopsy. At the time the sample was obtained, chemotherapy and radiotherapy treatment were already initiated. After implantation into mice, the tissue formed tumors that were used for the *in vivo* experiments.

PDX2: Patient derived tumors were established from a triple negative invasive ductal carcinoma from a female patient. The tissue was derived from a nodal recurrence at time of lymph node dissection. At the time the sample was obtained, chemotherapy and radiotherapy treatment were already initiated. After implantation into mice, the tissue formed tumors that were used for the *in vivo* experiments.

PDX304: The patient derived tumors were established from invasive ductal carcinoma of breast cancer, grade 3, with epidermoid features (metaplastic carcinoma) and foci of necrosis, involving upper outer quadrant and breast center, measuring 10 cm in largest dimension from a female patient. At the time the sample was obtained, taxol and cytoxan doxorubin treatment were already initiated. After implantation into mice, the tissue formed tumors that were used for the *in vivo* experiments.

PDX613: Patient derived tumors were established from TNBC from a female patient. The tissue was obtained by needle biopsy from a treatment naïve primary tumor. After implantation into mice, the tissue formed tumors that were used for the *in vivo* experiments.

Mass spectrometry.

Phosphotyrosine Analysis of Tumor Samples

Tumors were homogenized by sonication in 8M urea. Protein concentration was measured by a bicinchoninic acid assay (Pierce). Urea lysates were reduced with 10mM dithiothreitol (DTT), alkylated with 55mM iodoacetamide, and digested with trypsin overnight. Digested peptides were desalted using C18 cartridges (Waters) and labeled with TMT 10plex or TMTpro

isobaric mass tags (ThermoFisher Scientific). For the three runs utilizing untreated PDX tumors, a pooled normalization control containing peptides from each tumor was included to enable comparison of differentially abundant phosphotyrosine sites across PDXs in each mass spectrometry run. TMT-labeled peptide samples were subjected to a two-step enrichment process. Eluates were then subjected to LC-MS/MS on the Orbitrap Exploris 480 as follows: 1) Peptides were separated with a 140 min gradient with 70% acetonitrile in 0.2 M acetic acid. 2) The mass spectrometer was operated with a spray voltage of 2.5 kV. 3) Selected ions were HCD fragmented at normalized collision energy 33%. 4) Full MS1 scans were acquired in the m/z range of 380–2000, with maximum injection time determined automatically and data-dependent acquisition performed with a 3-second cycle time. 5) MS/MS acquisition was performed at a resolution of 60,000. Limited LC-MS/MS analysis of the most abundant peptides to adjust for channel-to-channel loading variation was carried out on an Orbitrap Q-Exactive Plus mass spectrometer using ~15 ng of peptide. Supernatant was loaded onto an acidified trapping column and analyzed with gradients as follows: 0%–13% solvent B in 4 minutes, 13%–42% in 46 minutes, 42%–60% in 7 minutes, 60%–100% in 3 minutes, and 100% for 8 minutes, before equilibrating back to Solvent A. Full scans (MS1) were acquired in the m/z range of 350–2000 at a resolution of 70,000 (m/z 100). The top 10 most intense precursor ions were selected and isolated with an isolation width of 0.4 m/z. Selected ions were HCD fragmented at normalized collision energy of 33% at a resolution of 70,000.

Phosphotyrosine Data Analysis

Raw mass spectral data files were processed with Proteome Discoverer version 3.0 (ThermoFisher Scientific) and searched against the human SwissProt database using Mascot version 2.8 (Matrix Science). Cysteine carbamidomethylation, TMT-labeled lysine, and TMT-labeled peptide N-termini were set as static modifications. Methionine oxidation and phosphorylation of tyrosine, serine, and threonine were searched as dynamic modifications. Peptide spectrum matches (PSMs) for phosphopeptides were filtered for ion score ≥ 20, search engine rank = 1, precursor isolation interference ≤ 35%, and average TMT intensity ≥ 1000. All remaining PSMs for a peptide with a given modification were summed to generate peptide-level TMT intensities. PSMs for the most abundant peptides in IP supernatant runs were filtered for ion score ≥ 25. Phosphopeptide TMT intensities were adjusted for sample loading variation using relative median values from IP supernatants run on the Q-Exactive Plus. For the three untreated PDX runs, PSMs with missing TMT values for all biological replicates for a given PDX line were included for PaSSS analysis, and these missing values were replaced with a value of 100. The relative abundance for each phosphopeptide was log2-transformed and visualized using MATLAB (version R2021b, Bioinformatics Toolbox version 4.15.2, MathWorks). Data were plotted with the 'clustergram' function with hierarchical clustering using Euclidean distance. Peptides that were present in at least two of the three untreated PDX runs were included in the heatmap for these samples. For visual clarity, PSMs with missing TMT values were not included in heatmaps.
Supplementary Tables S1-S3 and S4-S6 have a detailed list of the amino acid sequence of tyrosine phosphopeptides from each mass spec run and their abundances. The raw mass spectrometry data and associated tables have been deposited to the ProteomeXchange Consortium via the PRIDE partner repository with the dataset identifier PXD042640. Data can be accessed for manuscript review with the following credentials: Username: reviewer_pxd042640@ebi.ac.uk; Password: KCIWip8o .

Murine models

Allogeneic model: All the above-mentioned tumors were induced in NSG (Jackson Laboratory) female mice by transplanting xenografts orthotopically or injecting cells from pleural fluid.

After reaching the initial volume (60-80 mm^3), mice were randomly grouped to 8-10 animals per cage and treatment was initialized. Tumor sizes were routinely measured with an electronic caliper every two days and their volumes were obtained using the formula V = (W (2) × L)/2. Tumors were harvested after euthanizing mice. The time elapsed from tumor inoculation varied from 2 weeks to 1 month. All in-vivo experiments were performed with the approval of the Hebrew University of Jerusalem IACUC.

In vivo treatments

Targeted inhibitors: Erlotinib (EGFR inhibitor, #10483-1(Er)) and Trametinib (MEK inhibitor, #16292, (Tr)), AG-1024 (IGF-1R inhibitor, #14833-10) were purchased from Cayman Chemical. Taxol (#33069-62-4) was purchased from Teva Pharmaceutical Industries Ltd.
Erlotinib (12.5 mg/kg) and Trametinib (0.5 mg/kg) were given by gavage six consecutive days per week. The vehicle used was hydroxypropyl methylcellulose with 0.2% Tween. AG-1024 (1.5 mg/kg/six days per week) and Taxol (20 mg/kg/once a week) were given IP (vehicle was sterile saline). Mice were treated for 45 days.

Supplementary References:

1. Remacle F, Kravchenko-Balasha N, Levitzki A, Levine RD. Information-theoretic analysis of phenotype changes in early stages of carcinogenesis. Proc Natl Acad Sci U S A. 2010;107:10324–9.
2. McQuarrie DA (Donald A. Statistical mechanics. 2003;:641.
3. Edda Klipp; Wolfram Liebermeister; Christoph Wierling. Systems Biology: A Textbook. 2d edition. Wiley-Blackwell; 2016.
4. Kravchenko-Balasha N, Levitzki A, Goldstein A, Rotter V, Gross A, Remacle F, et al. On a fundamental structure of gene networks in living cells. Proc Natl Acad Sci U S A. 2012;109:4702–7.
5. Vasudevan S, Flashner-Abramson E, Remacle F, Levine RD, Kravchenko-Balasha N. Personalized disease signatures through information-theoretic compaction of big cancer data. Proc Natl Acad Sci U S A. 2018;115:7694–9.
6. Vasudevan S, Flashner-Abramson E, Alkhatib H, Roy Chowdhury S, Adejumobi IA, Vilenski D, et al. Overcoming resistance to BRAFV600E inhibition in melanoma by deciphering and targeting personalized protein network alterations. npj Precis Oncol. 2021;5:50.
7. Alkhatib H, Rubinstein AM, Vasudevan S, Flashner-Abramson E, Stefansky S, Chowdhury SR, et al. Computational quantification and characterization of independently evolving cellular subpopulations within tumors is critical to inhibit anti-cancer therapy resistance. Genome Med 2022 141. 2022;14:1–17.
8. Szklarczyk D, Franceschini A, Kuhn M, Simonovic M, Roth A, Minguez P, et al. The STRING database in 2011: functional interaction networks of proteins, globally integrated and scored. Nucleic Acids Res. 2011;39 Database issue:D561-8.
9. Szklarczyk D, Gable AL, Lyon D, Junge A, Wyder S, Huerta-Cepas J, et al. STRING v11: Protein-protein association networks with increased coverage, supporting functional

discovery in genome-wide experimental datasets. Nucleic Acids Res. 2019;47:D607–13.
10. Flashner-Abramson E, Vasudevan S, Adejumobi IA, Sonnenblick A, Kravchenko-Balasha N. Decoding cancer heterogeneity: Studying patient-specific signaling signatures towards personalized cancer therapy. Theranostics. 2019;9:5149–65.
11. Kreutzfeldt S, Horak P, Hübschmann D, Knurr A, Fröhling S. National Center for Tumor Diseases Precision Oncology Thesaurus for Drugs: A Curated Database for Drugs, Drug Classes, and Drug Targets in Precision Cancer Medicine. JCO Clin cancer informatics. 2023;7.
12. Petreus T, Cadogan E, Hughes G, Smith A, Pilla Reddy V, Lau A, et al. Tumour-on-chip microfluidic platform for assessment of drug pharmacokinetics and treatment response. Commun Biol 2021 41. 2021;4:1–11.
13. Paul Shannon 1, Andrew Markiel 1, Owen Ozier, 2 Nitin S. Baliga, 1 Jonathan T. Wang, 2 Daniel Ramage 2, Nada Amin 2, Benno Schwikowski, 1, 5 and Trey Ideker2, 3, 4 5, 山本隆久, et al. Cytoscape: A Software Environment for Integrated Models. Genome Res. 1971;13:426.
14. Kohale IN, Yu J, Zhuang Y, Fan X, Reddy RJ, Sinnwell J, et al. Identification of Src Family Kinases as Potential Therapeutic Targets for Chemotherapy-Resistant Triple Negative Breast Cancer. Cancers (Basel). 2022;14.
